# Identifying cytokine signaling signatures in primary human Th-1 cells by phospho-proteomics analysis

**DOI:** 10.1016/j.xpro.2021.100417

**Published:** 2021-03-31

**Authors:** Jonathan Martinez-Fabregas, Elizabeth Pohler, Ignacio Moraga

**Affiliations:** 1 Division of Cell Signaling and Immunology, School of Life Sciences, University of Dundee, Dundee, UK

**Keywords:** Cell isolation, Immunology, Signal Transduction, Protein Biochemistry, Proteomics

## Abstract

Stable isotope labeling by amino acid-based high-resolution phosphoproteomics is a powerful technique that allows for direct comparison of cells stimulated under different experimental conditions. This feature makes it the ideal methodology to identify cytokine signaling networks. Here, we present an optimized protocol for the isolation and identification of phosphopeptides from IL-6-stimulated primary human Th-1 cells.

For complete details on the use and execution of this protocol, please refer to Martinez-Fabregas et al. (2020).

## Highlights

• Phosphoproteomics protocol for studying cytokines signaling in immune cells
• Tips for the isolation and expansion of primary human Th-1 cells
• Detailed protocol for the efficient SILAC labelling of human Th-1 cells

## Graphical abstract

**Figure:**
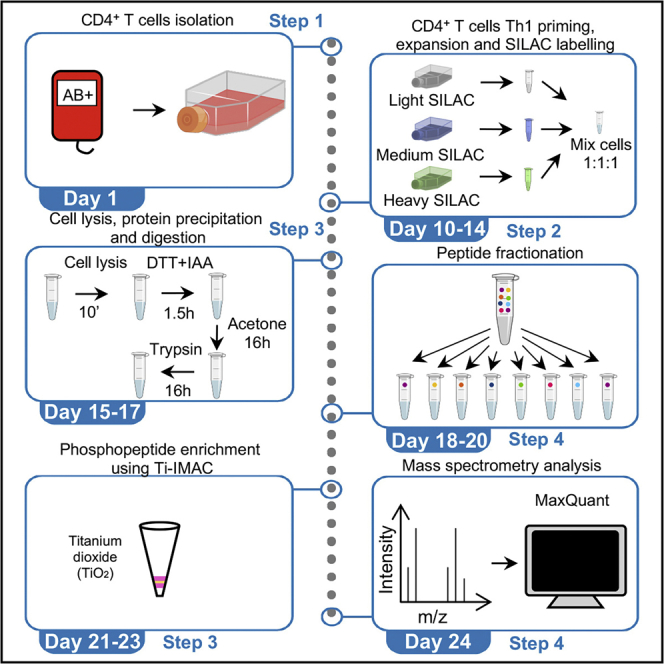

## Before you begin

This protocol describes the steps required for the isolation of phosphopeptides and for phosphoproteomics analysis in human primary T cells from healthy donors. Three different populations of T cells are generated by labeling them with light, medium, and heavy SILAC amino acids. This allows for side-by-side comparison of up to three different experimental conditions. All the work must be carried out in a Cat II hood. Cat II containers need to be ready to dispose of the blood residue and a 1% Virkon solution must be used to decontaminate all the materials that get in contact with the blood during the isolation of the cells.

## Key resources table

**Table:** 

REAGENT or RESOURCE	SOURCE	IDENTIFIER
**Antibodies**		

Rat anti-human-CD4-FiTC (clone A161A1)	BioLegend	Cat#357406; RRID: AB_2562357
Anti-FiTC microbeads	Miltenyi	Cat#130-048-701; RRID:AB_244371
Rat purified NA/LE anti-human-IL4 (clone MP4-25D2)	BD Biosciences	Cat#554481; RRID: AB_395421

**Biological samples**		

Human peripheral blood mononuclear cells	Scottish Blood Transfusion Service	N/A

**Chemicals, peptides, and recombinant proteins**		

Recombinant human interleukin-2	Novartis	Cat#709421
Recombinant human interleukin-12	BioLegend	Cat#573002
MSC2530818	Stratech	Cat#S8387-SEL
Recombinant human hyper-interleukin-6	Expressed and purified in the lab	N/A
ImmunoCult Human CD3/CD28 T Cell Activator	STEMCELL	Cat#10971
L-Lysine K0	Sigma	Cat#L8662
L-Arginine R0	Sigma	Cat#A8094
L-Proline P0	Sigma	Cat#P0380
L-Lysine U-13C6 K6	CKGAS	Cat##CLM-2247-0.25
L-Arginine U-13C6 R6	CKGAS	Cat#CLM-2265-0.25
L-Lysine U-13C6,U-15N2 K8	CKGAS	Cat#CNLM-291-H-0.25
L-Arginine U-13C6,U-15N2 R10	CKGAS	Cat#CNLM-539-H-0.25
RPMI SILAC media	Thermo Scientific	Cat#88365
Dialyzed FBS	HyClone	Cat#SH30079.03
L-Glutamine	Invitrogen	Cat#25030024
Pen/Strep	Invitrogen	Cat#15140122
MEM vitamin solution	Thermo Scientific	Cat#11120052
Selenium-transferrin-insulin	Thermo Scientific	Cat#41400045
Triethylammonium bicarbonate buffer	Thermo Scientific	Cat#90114
Sodium dodecyl sulfate	Sigma	Cat#71725
Dithiothreitol	Sigma	Cat#D0632
Iodoacetamide	Sigma	Cat#I6125
Trypsin	Thermo Scientific	Cat#90058
Trifluoroacetic acid LC-MS grade	Thermo Scientific	Cat#85183
Acetonitrile LC-MS grade	Thermo Scientific	Cat#51101
MagResyn Ti-IMAC	2BScientific	Cat#MR-TIM002
Ammonium hydroxide solution	Sigma	Cat#09859
Glycolic acid	Sigma	Cat#124737
Lymphoprep	STEMCELL	Cat#07801
LS columns	Miltenyi	Cat#130-042-401
QuadroMACS separator	Miltenyi	Cat#130-091-051

**Critical commercial assays**		

BCA Protein Assay Kit	Thermo Scientific	Cat#23227
Quantitative Colorimetric Peptide Assay	Thermo Scientific	Cat#23275

**Deposited data**		

Phosphoproteomics raw and analyzed data	Martinez-Fabregas et al., 2020	ProteomeXchange: PXDX020964

**Software and algorithms**		

MaxQuant	Cox and Mann, 2008	https://www.maxquant.org
Andromeda	Cox et al., 2011	https://www.maxquant.org
DAVID GO analysis tools	Huang et al., 2007; Huang da et al., 2009	https://david.ncifcrf.gov/home.jsp

## Materials and equipment

**CRITICAL:** Trifluoroacetic acid (TFA) is harmful when inhaled and is toxic to aquatic life. It needs to be used in an extraction hood to minimize the risk of inhalation and needs to be disposed following the appropriate health and safety regulations.

## Step-by-step method details

### Peripheral blood mononuclear cell (PBMC) isolation

**Timing: 3 h**

**Figure 1:**
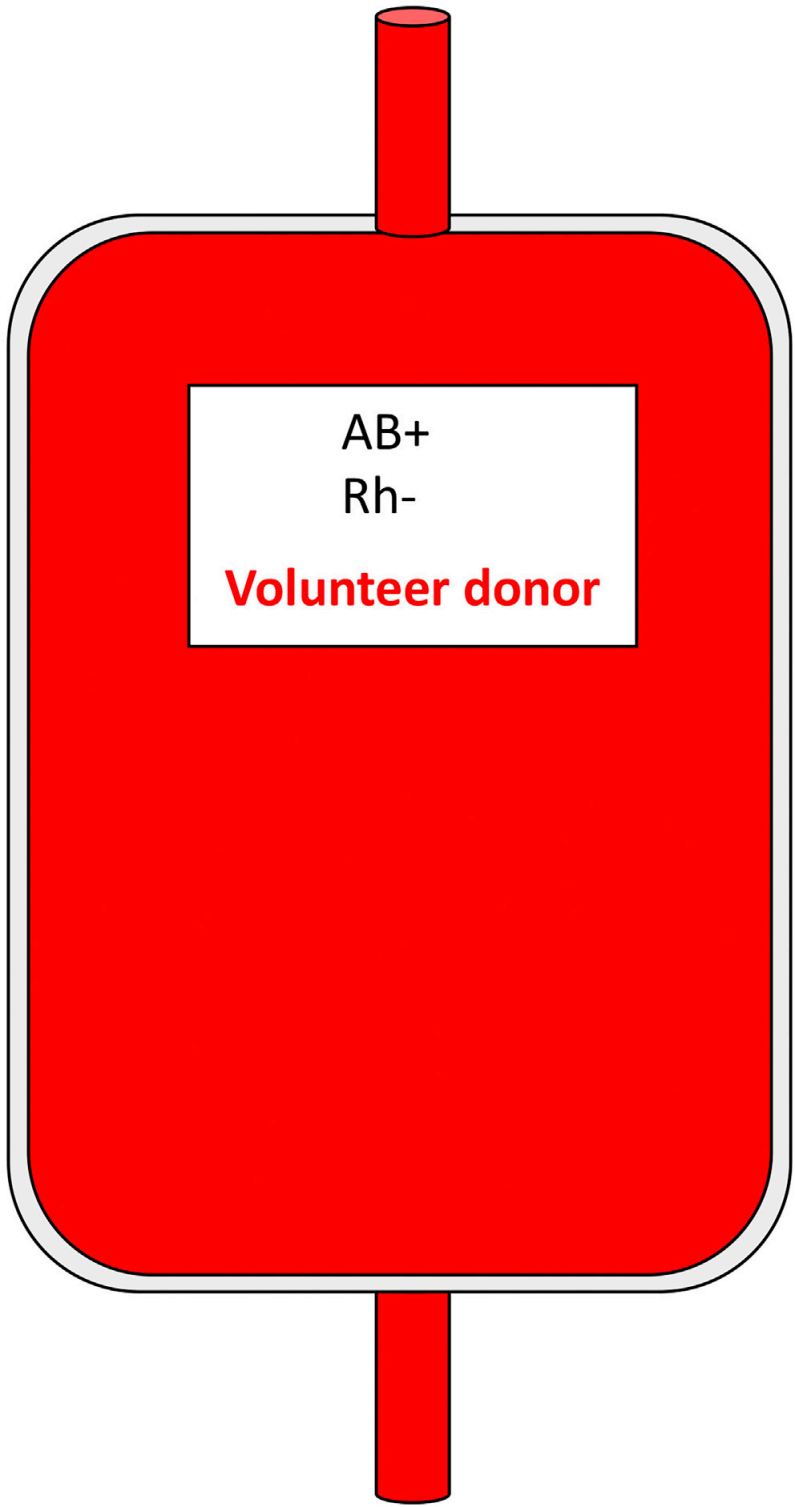
Blood transfusion bag scheme showing the upper and lower tubes for clarity
2. Add 25 mL of RPMI-1640 media without additives to each tube.
3. In 50 mL Falcon tubes add 25 mL of sterile Lymphoprep and slowly layer 25 mL of 1:2 diluted blood on top of the Lymphoprep layer.

***Note:*** Lymphoprep is a commercial density gradient medium recommended for the isolation of mononuclear cells from peripheral blood. Alternatively, Ficoll gradients can also be used for the isolation of lymphocytes (Corkum et al., 2015).

1. Blood donated by healthy donors was obtained from the Blood Transfusion Unit (Ninewells Hospital, Dundee, UK) already in blood transfusion bags (Figure 1). Cut the lower tube of the bag containing the blood samples. Make sure it is properly introduced in a 50 mL Falcon tube to avoid spillage of the blood and then cut the upper tube. Distribute the 50 mL of blood between two clean, sterile 50 mL Falcon tubes (25 mL in each tube).

**Figure 2:**
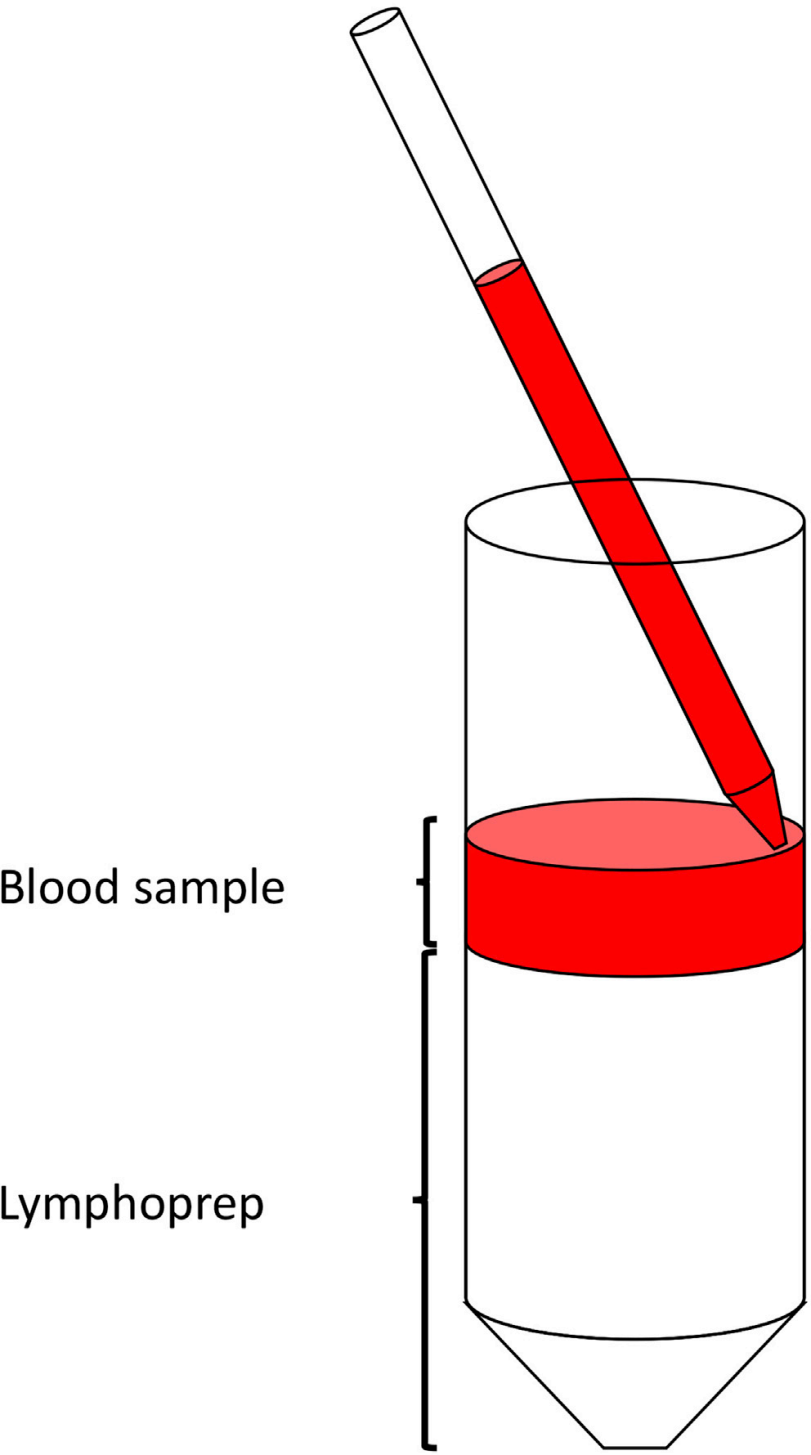
Schematic representation of the Blood-Lymphoprep layer preparation

**CRITICAL:** Special care is needed not to mix the Lymphoprep-Blood layers to get PBMCs clear of red blood cells. For that, use an electronic pipette controller with a 25 mL pipette. Carefully put the tip of the 25 mL pipette containing 25 mL of blood in touch with the Lymphoprep layer and slowly add the blood on top (Please refer to Figure 2).

**Figure 3:**
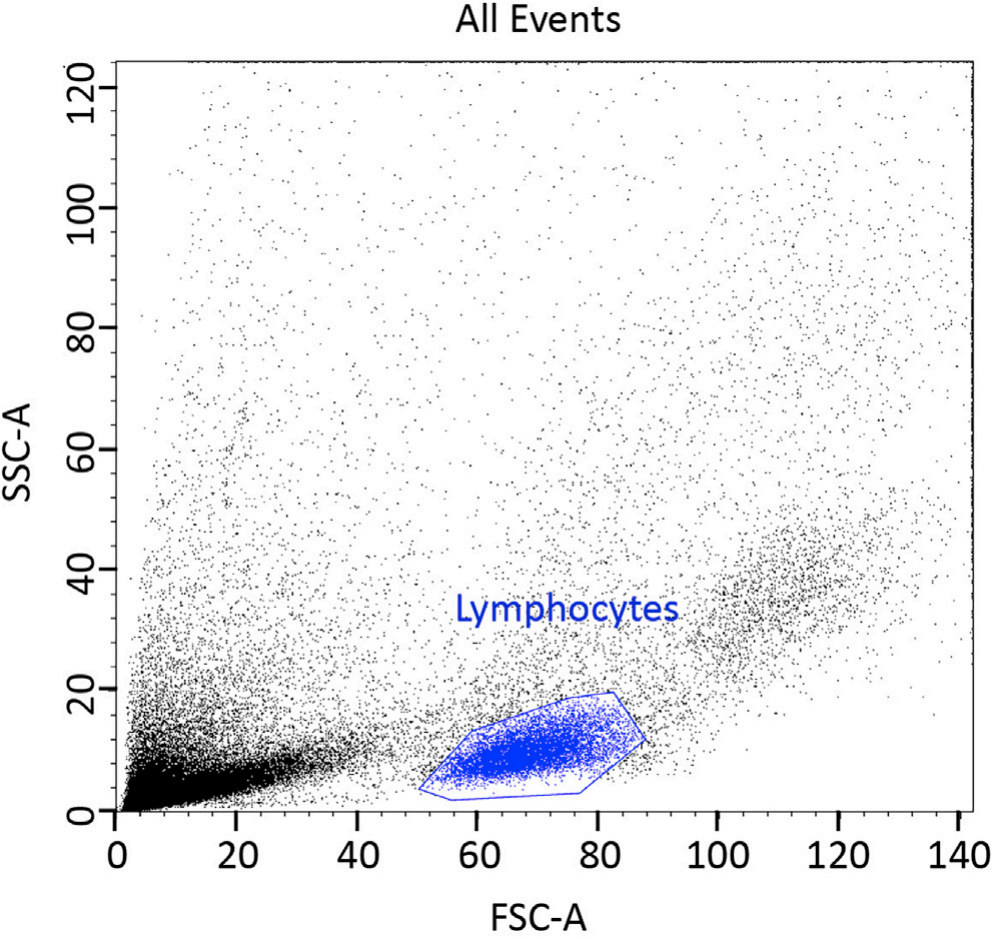
Lymphocytes gating using the forward and side scatter profile

4. Spin tubes at 800 *g* in a swing-bucket rotor centrifuge for 30 min at 18°C–25°C with maximum acceleration but with no brake.
5. Collect the white buffy coat layer that forms an interface between the plasma layer above and the red blood cells below into a clean, sterile 50 mL Falcon tube using sterile pastettes.
6. Wash the cells with 50 mL RPMI-1640 without additives and spin cells down cells by centrifugation: 5 min at 300 *g* maximum acceleration and maximum deceleration.
7. Repeat step 6 again.
8. Finally, resuspend the pellet in 50 mL of RPMI-1640 supplemented with 100 Units/mL Penicillin/, 100 μg/mL Streptomycin and 2 mM L-Glutamine.
9. Count the number of cells in a Flow cytometer gating on the lymphocytes based on their forward scatter (FSC) and side scatter (SSC) profile (Figure 3). Dilute 10 μL of the cell suspension obtained in step 8 in 490 μL RPMI (1:50 dilution) and count cells in the Flow cytometer for 60 s at a flow rate of 1 μL/s. Using the number of lymphocytes (Figure 2) counted in 60 s (60 μL) calculate the total number of lymphocytes in the 50 mL of sample obtained in step 8 taking into account the 1:50 dilution factor.

### Human primary CD4+ T cells isolation

**Timing: 3 h**

10. Transfer 10^8^ lymphocytes into a new 50 mL Falcon tube and spin them down by centrifugation at 300 *g* for 5 min.
11. Resuspend cells in 10 mL of sterile PBS supplemented with 0.5% BSA and 2 mM EDTA (PBE buffer).
12. Collect cells again by centrifugation at 300 *g* for 5 min.
13. Resuspend the cells in 500 μL PBE buffer (sterile PBS supplemented with 0.5% BSA and 2 mM EDTA), transfer to a 15 mL Falcon tube and stain them with 30 μL of anti-CD4-FiTC antibody at +4°C for 15 min.
14. Transfer 10 μL of stained cells into a 96 well V-bottom plate. This will be your pre-elution sample that will allow you to calculate the total number of positive CD4 T cells in the cell suspension and the efficiency of isolation.
15. Spin down cells in the Falcon tube at 300 *g* for 5 min at +4°C.
16. Repeat the PBE wash again to remove unbound antibody.
17. Resuspend the cells in 500 μL PBE buffer and add 50 μL of anti-FiTC microbeads.
18. Incubate the cells at +4°C for 15 min.
19. Resuspend the cells in 10 mL of PBE buffer and spin them down at 300 *g* for 5 min at +4°C.
20. Repeat step 19 twice to remove the excess of unbound microbeads.
21. Equilibrate an LS column on a QuadroMACS separator with 3 mL of PBE.
22. Resuspend the cell pellet in 3 mL of PBE buffer and proceed to the isolation of CD4 stained cells by Magnetic Activated Cell Sorting using the previously equilibrated LS column using a QuadroMACS separator.
23. After loading the cells onto the column wash the column three times with 3 mL of PBE buffer. Collect the eluates from the three washes plus the eluate after loading your cells as your flow-through samples. Transfer 10 μL of the flow-through into a 96 well V-bottom plate to check the efficiency of isolation at the end of the procedure.
24. Finally, remove the LS column containing the CD4 stained cells from the separator and place it into a 15 mL Falcon tube
25. Add 5 mL of PBE buffer and immediately flush out the CD4 positive T cells by firmly applying the plunger supplied with the LS column. This sample contains the CD4+ T cells. Transfer 10 μL of the flow-through into a 96 well V-bottom plate.
26. In the 96 well plate containing 10 μL from each sample add another 190 μL to dilute the samples (1:20) and count cell in a flow cytometer to check the efficiency of isolation and to determine the number of CD4+ T cells recovered at the end of the procedure. Add 190 μL of PBE to the 10 μL of samples in the 96 well plate (1:20 dilution) and count cells in a flow cytometer to check the efficiency of isolation and to determine the number of CD4+ T cells recovered at the end of the procedure.

***Note:*** Usually starting with 10^8^ lymphocytes, ~25–30 × 10^6^ CD4^+^ T cells with a purity greater than 95% should be recovered.

### Activation and SILAC labeling of cells

**Timing: 10–14 days**

27. Resuspend 10^7^ CD4+ T cells in 5 mL of RPMI-1640 supplemented with Pen/Strep and L-Glutamine and activate them for three days in the presence of 300 μL ImmunoCult Human CD3/CD28 T cell Activator and 20 ng/mL IL-2 under Th1 polarizing conditions: 20 ng/mL IL-12 and 10 ng/mL anti-IL-4.
28. After three days spin down cells at 300 *g* for 5 min and resuspend them in 10 mL of light, medium or heavy SILAC RPMI-1640 medium (please refer to Tables 1 and 2 in the materials and equipment section) supplemented with 20 ng/mL IL-2, 20 ng/mL IL-12 and 10 ng/mL anti-IL4.

**Table 1:** Preparation of SILAC amino acids stock solutions

Amino acid	Molecular weight	Stock (M)	Stock (1/1000)
Light L-Lysine (K0)	182	0.219	40 mg/ml
Light L-Arginine (R0)	174.2	0.48	84 mg/ mL
Medium L-Lysine 13C6 (K6)	225.1	0.219	49 mg/mL
Medium L-Arginine 13C6 (R6)	126.6	0.48	103 mg/mL
Heavy L-Lysine 13C6,15N2 (K8)	227.1	0.219	49.7 mg/mL
Heavy L-Arginine 13C6,15N4 (R10)	220.59	0.48	105.8 mg/mL
Light L-Proline (P0)	115.13	1.7	200 mg/ml

**Table 2:** Light, medium and heavy SILAC media preparation recipe

Reagent	Light SILAC media (K0+R0)	Medium SILAC media (K6+R6)	Heavy SILAC media (K8+R10)
SILAC RPMI-1640 media	500 mL	500 mL	500 mL
Dialyzed SILAC-grade FBS	50 mL	50 mL	50 mL
L-Glutamine 100×	5 mL	5 mL	5 mL
Penicillin/Streptomycin 100×	5 mL	5 mL	5 mL
MEM vitamin solution 100×	5 mL	5 mL	5 mL
Sodium pyruvate 100×	5 mL	5 mL	5 mL
Selenium, Transferrin & Insulin 100×	5 mL	5 mL	5 mL
Light L-Lysine (K0) stock	1× 0.5 mL aliquot		
Light L-Arginine (R0) stock	1× 0.5 mL aliquot		
Medium L-Lysine 13C6 (K6) stock		1× 0.5 mL aliquot	
Medium L-Arginine 13C6 (R6) stock		1× 0.5 mL aliquot	
Heavy L-Lysine 13C6,15N2 (K8) stock			1× 0.5 mL aliquot
Heavy L-Arginine 13C6,15N4 (R10) stock			1× 0.5 mL aliquot
Light L-Proline (P0)	1× 0.5 mL aliquot	1× 0.5 mL aliquot	1× 0.5 mL aliquot

***Note:*** Prepare amino acids stock solutions in newly opened, sterile PBS and store 0.5 mL aliquots at −20°C. Each aliquot is enough to prepare 500 mL of complete SILAC medium.

***Note:*** SILAC media for T cells is supplemented with light Proline as T cells can convert arginine into proline affecting the labelling, so by increasing the concentration of proline we block this pathway.

***Note:*** After combining all the reagents required for the preparation of SILAC media filter the media using a 500 mL Stericup Filtration System and keep the media at +4°C.

29. Count the number of cells every two-three days and dilute them down to 10^6^ cells / mL. Keep them growing in the appropriate SILAC media (light, medium or heavy) supplemented with IL-2, IL-12 and anti-IL-4 for 5 cell division cycles to ensure complete labeling.

### Preparation of sample for proteomics

**Timing: 2–3 days**

30. Count cells in a flow cytometer and collect about 10^8^ cells per SILAC labeling (light, medium or heavy) by centrifugation at 300 *g* for 5 min. Transfer them into a clean T175 flask containing 25 mL of the appropriate SILAC media without IL-2 and starve the cells for 24 h. Alternatively, at this stage cell counting could be done using standard cell counting practices, such as counting slides.

***Note:*** Cells are starved of cytokines (IL-2, IL-4 and IL-12) for 16 h before stimulation to reduce the basal levels of the signaling pathways engaged by IL-2, IL-4 and IL-12. This step allows us to reduce the confounding effects of having IL-2, IL-4 and IL-12 present during the stimulation with the cytokine of interest (i.e., IL-6 in this study).

31. Next day collect cells again by centrifugation, resuspend them in SILAC RPMI-1640 media containing FBS and stimulate them as required. In our case cells were treated as follows:
  a. T cells grown in light SILAC media were unstimulated and used as a reference control.
  b. T cells grown in medium SILAC media were stimulated with 20 nM IL-6 for 15 min.
  c. T cells grown in heavy SILAC media were treated with 2 μM of MSC2530818, a specific CDK8 inhibitor, for 30 min and then stimulated with 20 nM IL-6 for 15 min.
32. After stimulation, wash cells three times with 50 mL of ice cold PBS, then combine in a 1:1:1 (light : medium : heavy) ratio.

***Note:*** It is important to check for the label incorporation efficiency. To do so, collect about 10^6^ cells labelled with the different amino acids (light, medium, and heavy) and transfer them into a clean, low protein binding microcentrifuge tube. Follow the same protocol described below, just adjusting the volumes as required and analyze the peptides to determine the percentage of labelling of the different samples. At least, 95% labelling efficiency is required.

***Note:*** Light, medium and heavy SILAC labels are mixed at this point in order to avoid differences arising from the manipulation of the samples during cell lysis, protein precipitation and digestion, peptide fractionation and enrichment.

33. After the final wash, resuspend the cell pellet (1 volume) in SDS-lysis buffer (5 volumes) and incubate on ice for 10 min to ensure complete cell lysis.

***Note:*** SDS-containing lysis buffer should be freshly prepared by dissolving SDS (1% w/v) in 100 mM Triethylammonium Bicarbonate Buffer (TEAB) pH8.

34. Centrifuge the cell lysates at 20,000 *g* for 15 min at +4°C and transfer supernatants to clean Falcon tubes.
35. Determine protein concentrations using a BCA Protein Assay kit.
36. Transfer 10 mg of total protein per experiment of combined light, medium, and heavy labeled proteins into a clean, low protein binding microcentrifuge tube and reduce the protein sample with 10 mM dithiothreitol for 1 h at 55°C in order to break all disulfide bridges and linearize the proteins.
37. Alkylate the protein sample with 20 mM iodoacetamine for 30 min at 18°C–25°C in order to block the free cysteine residue and avoid the formation of new disulfide bridges.
38. Finally, precipitate the proteins using six volumes of chilled (−20°C) acetone for 16 h at −20°C.
39. After precipitation, protein pellets were obtained by centrifugation at 14,000 *g* for 30 min at +4°C . Then, resuspend protein pellet (10 mg) in 1 mL of 100 mM TEAB and digest with 100 μg of Trypsin (1:100 w/w) for 16–24 h at 37°C. Digestion was done in two steps. First add 50 μg of Trypsin to the protein suspension and incubate for 2 h. Then, add the remaining 50 μg of Trypsin and digest the protein for 16–24 h at 37°C in a thermoblock with shaking at 1000 rpm.

***Note:*** After resuspension in 1 mL of 100 mM TEAB the protein suspension can look cloudy as proteins will not be completely in solution. This is normal and the protein solution will become clear during protein digestion.

***Note:*** Trypsin purchased from Thermo in 100 μg vials was reconstituted using 50 mM acetic acid to (1 mg / mL) (i.e., add 100 μL of 50 mM acetic acid to 100 μg of lyophilized Trypsin).

40. After protein digestion, clear the samples by centrifugation at 20,000 g for 30 min at +4°C, and determine the peptide concentration using a Quantitative Colorimetric Peptide Assay.
41. Lyophilize 3.5 mg of peptides per sample (already containing the light, medium, and heavy peptides) using an EZ-2 Plus Centrifugal Evaporator (GeneVac, HPLC program, 40 h, 40°C).

### Peptide fractionation

**Timing: 5–6 h per sample**

***Note:*** This step is not required to determine the labelling incorporation efficiency, so samples prepared to check this can be already analyzed by mass spectrometry and .RAW files analyzed by MaxQuant (see below).

42. Resuspend the lyophilized peptide samples in 200 μL Buffer A (10 mM ammonium formate), trap them on a XBridge trap cartridge (Waters, C18, 3.5 μM, 4.6 × 20 mm) and separate them on a XBridge Peptide BEH column (Waters, C18, 3.5 μM, 4.6 × 250 mm) using an Ultimate 3000 RSLCnano system (Thermo Scientific). Resolve peptides using a gradient (102 min, 0.8 mL/min) of Buffer A (10 mM ammonium formate) and Buffer B (10 mM ammonium formate, 90% acetonitrile): 8% Buffer B for 16 min, 8%–45% Buffer B for 54 min, 45%–100% Buffer B for 5 min, 100% Buffer B for 16 min and 100%–2% Buffer B for 21 min.
43. Collect 80 fractions of 0.8 mL each (1 min) using a WPS-3000FC autosampler (Thermo Scientific) from 1 to 80 min over the chromatogram.
44. Concatenate fractions to 20 fractions to provide a similar quantity of peptide per fraction based on the online (U3000 Variable Wavelength Detector, Thermo Scientific) UV values of the eluted peptides at 200 nm.
45. Dry fractions (EZ-2 Plus Centrifugal Evaporator, GeneVac) prior to phosphopeptide enrichment.

### Phosphopeptide enrichment

**Timing: depends on the number of samples to be processed; in our case 2–3 days**

***Note:*** Buffers required for phosphopeptide enrichment and purification are described in Table 3.

46. For phosphopeptide enrichment, use 50 μL (1 mg of beads) of MagResyn Ti-IMAC beads per sample to ensure complete depletion of phosphopeptides. Phosphopeptide enrichment could be done using an automatic processing pipeline (Tape et al., 2014).
47. For this protocol 60 fractions of peptides (20 fractions per replica) were being used. Thus, transfer 3 mL of MagResyn (50 μL per sample) into 2 mL clean low protein binding microcentrifuge tube (1.5 mL per tube).
48. Place tubes in a magnetic separator, and after 10–20 s remove the solution.
49. Resuspend particles in 2 mL of Loading buffer and allow 1 min for equilibration.
50. Place tubes back in the magnetic separator allowing 10–20 s for microparticles to clear. Then, remove the loading buffer.
51. Repeat steps 49 and 50 three more times.
52. After the last step of equilibration distribute microparticles into 60 1.5 mL clean, low protein binding microcentrifuge tubes.
53. Place tubes in a magnetic separator and remove supernatant. Microparticles are now ready for the phosphopeptide enrichment procedure.
54. Resuspend each peptide fraction in 100 μL of loading buffer and vortex to allow complete resuspension of the peptides.
55. Centrifuge samples for 10 min at 15000 *g* to remove insoluble material and transfer the supernatant into 1.5 mL clean, low protein binding microcentrifuge tubes containing the MagResyn Ti-IMAC microparticles, resuspending the microparticles by vortexing samples for 10 s.
56. Incubate the peptide – microparticles mixture for 20 min at 18°C–25°C with continuous mixing.

**CRITICAL:** In order to optimize the binding of the phosphopeptides to the TiO2 microparticles it is essential to apply continuous mixing (every 2–3 min, slow vortexing) during the incubation at 18°C–25°C.

57. Place the tube on the magnetic separator, allow 10–20 s for the magnetic beads to separate. Remove and discard the supernatant.

***Note:*** This supernatant should contain unbound, non-phosphorylated peptides.

58. Wash the microparticles with 100 μL of loading buffer for 2 min with gentle shaking in order to remove unbound peptides. After this, place the tubes again in the magnetic separator and once the microparticles have cleared remove and discard the supernatant.
59. Wash the microparticles with 100 μL of Wash buffer 1 for 2 min with gentle shaking in order to remove peptides bound non-specifically.
60. Remove the supernatant by placing the tubes back in the magnetic separator and allow 10–20 s for the magnetic microparticles to clear.
61. Repeat steps 59 and 60 for an additional wash discarding the supernatant after every wash.
62. Wash the microparticles for 2 min with 100 μL of Wash buffer 2. After incubation place the tubes back in the magnetic separator, remove, and discard the supernatant.
63. Resuspend the magnetic microparticles with 150 μL of Elution buffer and incubate for 10 min at 18°C–25°C to allow the elution of the phosphopeptides.

**CRITICAL:** In order to optimize the elution of the phosphopeptides from the magnetic microparticles it is essential to ensure the microparticles remain in suspension by constant gentle agitation in a shaker at 1000 rpm during the elution.

64. Recover the phosphopeptides in suspension by placing the tubes in the magnetic separator for 10 s to allow the magnetic microparticles to clear. After this, transfer the supernatant (eluted phosphopeptides) to a clean 1.5 mL protein low protein binding microcentrifuge tube with 50 μL of formic acid 10%.
65. To maximize phosphopeptide recovery repeat the elution steps 62 and 63 with another 150 μL of Elution buffer.
66. Place the tubes again in the magnetic separator, allow 20 s for microparticles to clear and pool the supernatant with the previously eluted phosphopeptides, adding a further 50 μL of 10% formic acid for a total volume of 400 μL.
67. Vacuum dry the eluates using an EZ-2 Plus Centrifugal Evaporator (GeneVac, HPLC program, 40 h, 40°C).

### Mass spectrometry analysis of phosphopeptides

**Timing: depends on the number of samples to be processed; in our case 1 week**

68. Phosphopeptide samples were analyzed using a nanoflow liquid chromatography system (Ultimate 3000 RSLCnano, Thermo Scientific) coupled to a Q Exactive Plus Mass Spectrometer (Thermo Scientific).

***Note:*** Mass spectrometry analysis can be carried out using different system (e.g., Q Exactive HF, LTQ Orbitraps, etc)

69. Phosphopeptide samples (10 μL) were loaded onto a C18 trap column and washed for 5 min with 0.1% formic acid.
70. Phosphopeptides were resolved using a gradient (170 min, 0.3 μL/min) of Buffer A (0.1% formic acid) and Buffer B (80% acetonitrile in 0.08% formic acid): 5% Buffer B for 5 min, 5%–35% Buffer B for 125 min, 35%–98% Buffer B for 2 min, 98% Buffer B for 20 min, 98%–2% Buffer B for 1 min and 2% Buffer B for 17 min.
71. Phosphopeptides, initially trapped in an Acclaim PepMap 100 C18 column (100 μm x 2 cm, Thermo Scientific), were separated on an Easy-Spray PepMap RSLC C18 column (75 μm x 50 cm, Thermo Scientific)
72. Finally, the phosphopeptides were transferred to a Q Exactive Plus Mass Spectrometer via and Easy-Spray source with temperature set at 50°C and a source voltage of 0.2 kV.
73. Peptides were identified with a top 15 method (1 MS plus 15 MS^2^, 150 min acquisition) consisting of full scans and mass range (m/z) between 350 to 1600 (m/z) for MS search and 200 to 2000 (m/z) for MS^2^ search.
74. For the MS^2^ scan the Q Exactive Plus Mass Spectrometer was operated in a data dependent acquisition mode, resolution of 70,000 with a lock mass set at 445.120024 and max fill time of 20 ms.
75. For the MS^2^ scan the Q Exactive Pluss Mass Spectrometer was operated in a centroid mode, resolution of 15,000 with isolation window = 1.4 (m/z), normalized collision energy = 27, max fill time of 100 ms and dynamic exclusion of 45 s.

### Analysis of mass spectrometry raw files

**Timing: depends on the number of samples to be processed; in our case 1**–**2 week**

76. Q Exactive Plus Mass Spectrometer .RAW files were analyzed and phosphopeptides identified and quantified using MaxQuant software (Cox and Mann, 2008), utilizing the built-in search engine Andromeda (Cox et al., 2011).

***Note:*** There are available different proteomic software for phosphopeptide identification and quantitation (e.g., PEAKS (Bioinformatics Solutions), Proteome Discoverer (Thermo), Scaffold (Proteome Software)).

77. All settings were set as default, except for minimal peptide length of 5, and the Andromeda search engine was configured for the Uniprot Homo sapiens database (release date: 2018_09).
78. Only phosphopeptides quantified in at least two out of three replicates were considered, and the p values were determined by Student’s t test corrected for multiple testing using the Benjamini–Hochberg procedure (Benjamini and Hochberg, 1995).
79. GO analysis were carried out using DAVID GO analysis tools (Huang da et al., 2009; Huang et al., 2007).

## Expected outcomes

Upon activation and expansion of 10^7^ CD4+ T cells in SILAC media for 5 cell division cycles more than 10^8^ SILAC-labeled cells should be recovered. Upon stimulation with the different experimental conditions we only combined 10^8^ cells per condition (light, medium, and heavy SILAC labeling) as those cell numbers (3 × 10^8^ total cells) should render enough protein (roughly 30 mg of protein) to repeat the experiment if something fails. After protein reduction with 10 mM DTT and alkylation with 20 mM iodoacetamide protein was precipitated for 16 h with six volumes of chilled acetone at −20°C. After protein precipitation, the protein pellet can be easily resuspended in 1 mL of 100 mM TEAB although it will appear cloudy. This is to be expected as proteins do not completely go into solution. The protein suspension will become clear upon digestion with Trypsin.After protein digestion the quantification of peptide concentration in all the samples is recommended before proceeding to peptide fractionation, phosphopeptide enrichment, and mass spectrometry analysis. In our hands, after protein precipitation and digestion we were able to recover about 7 mg of peptides that were then used for peptide fractionation into 20 individual fractions each of them containing roughly 350 μg of peptides. These peptide fractions were used for phosphopeptide enrichment then were analyzed by mass spectrometry and finally quantified using MaxQuant (Cox and Mann, 2008; Cox et al., 2011). This experimental approach normally allows for the identification and quantification of roughly 20,000 unique phosphopeptides.

## Limitations

SILAC-based proteomic and phosphoproteomic studies relies on the complete incorporation of the SILAC amino acids into the proteins (Mann, 2006; Ong et al., 2002). For that reason, cells need to proliferate and divide for at least 3 cell division cycles, somehow limiting the application of SILAC based proteomics and phosphoproteomic studies to cell types that can be expanded *in vitro* in SILAC media. Thus, checking the labeling incorporation is essential in order to guarantee the success of the study. If complete labeling cannot be achieved, then alternative methods (e.g., label-free or TMT-labeling techniques) (Kauko et al., 2015; Navarrete-Perea et al., 2018; Zecha et al., 2019; Zhang et al., 2015) need to be used. Additionally, due to the high number of cells needed for phosphoproteomic studies, its application in rare cell populations would be challenging.

## Troubleshooting

### Problem 1

A low number of lymphocytes (less than 10^8^) were obtained after PBMCs isolation.

### Potential solution

Make sure that the white buffy coat layer is completely collected to maximize the recovery PBMCs (step 5). However, sometimes this is due to poor quality of the blood sample obtained (e.g., not a fresh sample of blood). In these circumstances a new, fresh sample of blood is required to start the protocol again.

### Problem 2

A low number of CD4^+^ T cells were obtained after isolation (Step 26).

### Potential solution

Check your flow-through samples for the presence of unbound FiTC-positive CD4^+^ T cells. If this happens add another 50 μL of anti-FiTC microbeads and repeat the elution again to recover all the FiTC-positive CD4^+^ T cells.

If the flow-through is depleted of FiTC-positive CD4^+^ T cells start from a larger number of lymphocytes.

### Problem 3

Purity of Isolated CD4^+^ T cells was less than 95% (Step 26).

### Potential solution

Increase the number of washes with 3 mL of PBE buffer before the elution step.

### Problem 4

SILAC labeling checks show an amino acid labeling incorporation less than 95% (Step 31).

### Potential solution

Even though 5 cell division cycles should give a labeling incorporation efficiency greater that 98%–99%, if labeling incorporation is smaller than 95%, CD4+ T cells can be kept growing in SILAC media for an extra cell division cycle.

### Problem 5

Low recovery or low signal after phosphopeptide enrichment.

### Potential solutions

Low recovery of phosphopeptides (Step 63) during the phosphopeptide enrichment could be due to the use of a incorrect elution buffer. Make sure the concentration of NH_4_OH is 1%. Also the elution of phosphopeptides can be maximized by increasing the volume of elution buffer used or alternatively by increasing the number of elution steps. Low signal of phosphopeptides (Step 68) can be solved by concentrating the phosphopeptide samples by lyophilization prior to MS analysis.

## Resource availability

### Lead contact

Further information and requests for resources and reagents should be directed to and will be fulfilled by the lead contact Ignacio Moraga Gonzalez (imoragagonzalez@dundee.ac.uk).

### Materials availability

This study did not generate new unique reagents.

### Data and code availability

The datasets generated during this study have been deposited in the ProteomeXchange: PXD020964 and are described in (Martinez-Fabregas et al., 2020).

**Table 3:** Buffers required for the phosphopeptide enrichment and purification

Reagent	Composition
Loading buffer	0.1 M glycolic acid in 80% acetonitrile (ACN) and 5% trifluoroacetic acid (TFA)
Wash buffer 1	80% ACN, 1% TFA in mass spectrometry grade H_2_O
Wash buffer 2	10% ACN, 0.2% TFA in mass spectrometry grade H_2_O
Elution buffer	1% NH_4_OH

All these buffers should be freshly made.
